# Association of periodontal therapy, with inflammatory biomarkers and complications in COVID-19 patients: a case control study

**DOI:** 10.1007/s00784-022-04631-6

**Published:** 2022-07-29

**Authors:** Khalid N. Said, Ahmed M. Al-Momani, Jassim A. Almaseeh, Nadya Marouf, Amer Shatta, Jassim Al-Abdulla, Sahar Alaji, Hanin Daas, Shailaja S. Tharupeedikayil, Venkateswara Rao Chinta, Ali Ait Hssain, Mohammad Abusamak, Shiraz Salih, Noha Barhom, Wenji Cai, Mariano Sanz, Faleh Tamimi

**Affiliations:** 1 Department of Dentistry and Oral Health Institute, Hamad Medical Corporation, Doha, Qatar; 2 College of Dental Medicine, QU Health, Qatar University, Doha, Qatar; 3 Department of Business Intelligence, Hamad Medical Corporation, Doha, Qatar; 4 Medical Intensive Care Unit, Hamad Medical Corporation, Doha, Qatar; 5 Faculty of Dentistry, McGill University, Montreal, Canada; 6 Faculty of Odontology, ETEP Research Group, Universidad Complutense de Madrid, Madrid, Spain

**Keywords:** Periodontitis, Periodontal disease, COVID-19, Periodontal therapy, D-dimer

## Abstract

**Background:**

In previous studies, COVID-19 complications were reported to be associated with periodontitis. Accordingly, this study was designed to test the hypothesis that a history of periodontal therapy could be associated with lower risk of COVID-19 complications.

**Methods:**

A case–control study was performed using the medical health records of COVID-19 patients in the State of Qatar between March 2020 and February 2021 and dental records between January 2017 and December 2021. Cases were defined as COVID-19 patients who suffered complications (death, ICU admissions and/or mechanical ventilation); controls were COVID-19 patients who recovered without major complications. Associations between a history of periodontal therapy and COVID-19 complications were analysed using logistic regression models adjusted for demographic and medical factors. Blood parameters were compared using Kruskal–Wallis test.

**Results:**

In total, 1,325 patients were included. Adjusted odds ratio (AOR) analysis revealed that non-treated periodontitis was associated with significant risk of need for mechanical ventilation (AOR = 3.91, 95% CI 1.21–12.57, *p* = 0.022) compared to periodontally healthy patients, while treated periodontitis was not (AOR = 1.28, 95% CI 0.25–6.58, *p* = 0.768). Blood analyses revealed that periodontitis patients with a history of periodontal therapy had significantly lower levels of D-dimer and Ferritin than non-treated periodontitis patients.

**Conclusion:**

Among COVID-19 patients with periodontal bone loss, only those that have not received periodontal therapy had higher risk of need for assisted ventilation. COVID-19 patients with a history of periodontal therapy were associated with significantly lower D-dimer levels than those without recent records of periodontal therapy.

**Clinical relevance:**

The fact that patients with treated periodontitis were less likely to suffer COVID-19 complications than non-treated ones further strengthen the hypothesis linking periodontitis to COVID-19 complications and suggests that managing periodontitis could help reduce the risk for COVID-19 complications, although future research is needed to verify this.

**Supplementary information:**

The online version contains supplementary material available at 10.1007/s00784-022-04631-6.

## Introduction

The coronavirus disease of 2019 (COVID-19) caused by the severe acute respiratory syndrome coronavirus 2 (SARS Cov-2) has compromised the well-being of individuals and healthcare systems worldwide [1, 2]. COVID-19 is mostly mild or asymptomatic, but, in some patients, it can cause severe complications and even death [3, 4]. These complications are associated with an exacerbated inflammatory response called the “cytokine storm”, which is characterized by lymphocytopenia, neutrophilia, coagulopathies and increased blood levels of inflammatory cytokines [5–7]. COVID-19 complications are associated to certain risk factors (i.e. age, gender (male), obesity, hypertension, cardiovascular disease (CVD), cerebrovascular disease (CVD), chronic kidney disease and diabetes [8]) that have also been linked to periodontitis [9]. This has prompted the study of a possible association between periodontitis and COVID-19 severity [10]. Indeed, a study from our group has shown a significant association between periodontal bone loss and increased risk of COVID-19 complications [11], other groups have reported that poor oral health correlates significantly with COVID-19 severity [12, 13], and another study has shown that periodontitis could exacerbate the negative impact of obesity on COVID-19 [14].

Besides the association between periodontitis and the medical conditions described above, intervention trials and observational studies have also demonstrated that periodontal therapy may significantly reduce the risk of these conditions. For instance, recent systematic reviews have shown a reduction in HbA1c and fasting plasma glucose after periodontal treatment and could prevent diabetic complications [9, 15]. Similarly, randomized controlled trials have shown that periodontal therapy can reduce serum inflammatory mediators and induce positive changes in surrogate measures of atherothrombosis [16]. Moreover, recent population-based studies using medical registries have demonstrated that oral care interventions such as frequent tooth brushing and professional cleaning were associated with lower cardiovascular risk [17].

These associations have led us to hypothesize that periodontal care could also play a role in preventing COVID-19 complications. It was, therefore, the purpose of this study to evaluate the impact of periodontal treatment on the occurrence of COVID-19 complications.

## Material and methods

Ethical approval for this retrospective case–control study was obtained from the Institutional Review Board of Hamad Medical Corporation (HMC) with a waiver of informed consent under the pandemic response framework (protocol number MRC-01–20-1228). The study was conducted according to the STROBE guidelines.

### Study population

This is an expansion on our previous study in terms of scope and sample size [11]. Adult subjects (18 years and older) were selected from the electronic records of HMC based on a confirmed COVID‐19 diagnosis between March, 1st 2020, and December, 31st, 2020. To assess the disease outcome, COVID-19 data on these patients was extracted until February 28th, 2021. Both hospitalized and out-patient records were included.

HMC is the sole provider of medical care for COVID-19 patients in Qatar, a service provided free of charge to all patients in the country. Moreover, in Qatar, regular medical and dental care are offered free of charge for citizens and at significantly low cost for non-citizens in the public healthcare system. The medical and dental electronic records are linked through a unique hospital identification number for every patient.

All COVID-19 patients with active medical and dental records at HMC, including panoramic radiographs taken between January, 1st, 2017, and December, 31st, 2021, were included. Exclusion criteria were young age (less than 18 years of age) and lack of dental records. The COVID-19 vaccination records of the country were also searched to assess the vaccination status of the patients prior to their COVID-19 infection.

### Study design

Cases were defined as patients that experienced COVID‐19-related complications such as ICU admission, mechanical ventilation and/or death. Controls were COVID‐19 patients that recovered without major complications. All controls were included for analysis and no matching was performed.

All medical data from cases and controls including COVID-19 outcomes, predisposing conditions and blood sample analysis were extracted from HMC electronic records as described previously [11]. Information on predisposing conditions included patient’s sex, age, body mass index (BMI, kg/m^2^), smoking habits, asthma, chronic respiratory diseases, chronic heart disease, diabetes, dermatitis, chronic liver disease, autoimmune diseases, solid organ transplant, peptic ulcer, immunosuppressive conditions, cancer, chronic kidney disease, hypertension, cerebrovascular accidents and deep vein thrombosis.

Blood biomarkers analysed included D‐dimer, C-reactive protein (CRP), urea, creatinine, ferritin, interleukin-6 (IL-6), HbA1c, vitamin D, white blood cells (WBC) and lymphocytes. Both the initial blood tests taken upon COVID-19 diagnosis and the latest parameters measured prior to discharge were included for both cases and control patients.

The periodontal status was assessed on patient's panoramic radiographs by measuring inter-dental bone loss in posterior teeth using the criteria of the 2017 classification of periodontal diseases [18]. Periodontal bone loss was defined as bone loss at two or more non‐adjacent teeth after excluding local factors (periodontal‐endodontic lesions, cracked and fractured roots, caries, restorative factors and impacted third molars). Given the low sensitivity of panoramic radiographs [19], patients were categorized into two groups [20]: (i) non-to-mild periodontal bone loss (stages 0–1): bone loss ≤ 15% of the root length; and (ii) moderate-to-severe periodontal bone loss (stages 2–4): bone loss > 15% of the root length. To assess differences in bone loss severity across groups, we compared the actual percentage of inter-dental bone loss at posterior teeth. Each radiograph was assessed by two blinded investigators. In case of discrepancy, a third blinded investigator reviewed the radiographs, and the majority diagnosis was considered. All examiners were calibrated before the beginning of the study using two sets of 20 radiographs each, 14 days apart reaching a kappa index of 85%.

Patients were considered to have received periodontal treatment if they had radiographic signs of periodontal bone loss and at least one periodontal therapy procedure documented in their records for either non-surgical (i.e., scaling, root planing and polishing) or surgical periodontal treatment (i.e., periodontal flap procedure, regenerative therapy, osseous surgery, gingivectomy) between January, 1st, 2019, and December, 31st, 2020. Only periodontal treatments conducted before the subject was diagnosed with COVID-19 infection were considered for this investigation. To validate the radiographic assessment of periodontitis and the effectiveness of periodontal treatments, we analysed the subset of dental records within the cohort of patients followed at the Division of Periodontics at HMC. Response to periodontal therapy was reported in the dental records as changes in probing depth, reduced bleeding on probing, improved attachment level and reduced plaque levels.

Hospitalized patients with COVID-19 were assessed for respiratory bacterial infections if clinical signs of infection were compatible with laboratory and imaging findings. Respiratory specimens were collected by either endotracheal aspirate or bronchoalveolar lavage, and bacterial pathogens were defined using standard culture methods.

### Data analysis

Sample size was calculated to obtain 80% power with α-error of 0.05, and effect size *f*^2^ of 0.10 and 10 predictors based on our previous study [11]. An estimated sample size of at least 172 subjects per group was found to be needed to compare COVID-19 complications between periodontally healthy and patients with periodontal bone loss who were not treated for periodontitis.

The association between periodontal bone loss, periodontal therapy and COVID‐19 severity was analysed using logistic regression models adjusted for confounders including age, sex, smoking, BMI, citizenship, diabetes and co-morbidities. Age was used as a continuous variable, BMI was categorized as overweight/obese (BMI ≥ 25) and adequate/underweight (BMI < 25), and smoking was categorized as current/past and never smokers. Given the low prevalence of other comorbidities, we created a comorbidity score as proposed elsewhere [21]. This comorbidity score included hypertension, asthma, chronic respiratory disease, chronic heart disease, dermatitis, chronic liver disease, common autoimmune diseases (rheumatoid arthritis, systemic lupus erythematosus or psoriasis), solid organ transplant, peptic ulcer, immunosuppressive conditions, cancer, chronic kidney disease, cerebrovascular accident and deep vein thrombosis. For these conditions, a variable “comorbidity” was generated by computing the presence of each of the above conditions according to the number of comorbidities into 0, 1 and ≥ 2. This comorbidity index was validated using an OR and Chi-square test, and it showed to be a very strong predictor of COVID-19 complications (Supp. Table 6). Logistic regression analyses were reported as adjusted odds ratios (AOR) with 95% confidence intervals (CIs). Periodontal bone loss (%) across groups was compared using two-way ANOVA with post hoc Bonferroni correction. Laboratory biomarkers were assessed for normality and compared across groups using Kruskal–Wallis test with Bonferroni correction for multiple testing. Differences were considered statistically for *p* < 0.05. Statistical analyses were done using SPSS (v20.0, SPSS, IBM, Armonk, NY).

## Results

Out of the 141,422 COVID-19 cases registered in Qatar by end of February 2021, 2,621 had active dental records; among these patients, 1247 were excluded for not having recent panoramic radiographs and 49 for not having posterior teeth suitable for periodontal assessment. Thus, 1,325 patients fulfilled the above-mentioned criteria and were included for analysis; none of these patients were vaccinated by the time of their SARS-CoV-2 infection. From these patients, 71 suffered severe COVID-19 complications resulting in ICU admission, mechanical ventilation and/or death. Patients with complications were more likely to be males, older, and suffering from comorbidities (Table 1).

**Table 1:** Description of the study cohort according to the demographics, medical conditions and COVID-19 complications

Conditions	No complications (*n* = 1254)	Complications (*n* = 71)	*p*
Age (median [IQR])	39.00 [30.00, 52.00]	50.00 [41.50, 61.50]	** < 0.001** ^a^
BMI (median [IQR])	29.03 [25.22, 32.86]	29.88 [24.07, 35.48]	0.488 ^a^
Sex (%)			
Female	718 (57.2)	30 (42.3)	**0.014** ^b^
Male	536 (42.8)	41 (57.7)	
Citizenship status (%)			
Non-Qatari citizen	573 (45.7)	40 (56.3)	0.080 ^b^
Qatari citizen	681 (54.3)	31 (43.7)	
Smoking habits (%)			
Never smoker	1090 (86.9)	59 (83.1)	0.368 ^b^
Past/present smoker	164 (13.1)	12 (16.9)	
Hypertension (%)			
No	972 (77.5)	31 (43.7)	** < 0.001** ^b^
Yes	282 (22.5)	40 (56.3)	
Diabetes (%)			
No	894 (71.3)	29 (40.8)	** < 0.001** ^b^
Yes	360 (28.7)	42 (59.2)	
Asthma (%)			
No	1091 (87.0)	51 (71.8)	**0.001 **^b^
Yes	163 (13.0)	20 (28.2)	
Autoimmune (%)			
No	1237 (98.6)	71 (100.0)	0.323 ^b^
Yes	17 (1.4)	0 (0.0)	
Cardiovascular (%)			
No	1018 (81.2)	27 (38.0)	** < 0.001** ^b^
Yes	236 (18.8)	44 (62.0)	
COPD (%)			
No	1247 (99.4)	68 (95.8)	**0.001** ^b^
Yes	7 (0.6)	3 (4.2)	
Peptic ulcer (%)			
No	1230 (98.1)	67 (94.4)	**0.034** ^b^
Yes	24 (1.9)	4 (5.6)	
Cancer (%)			
No	1233 (98.3)	57 (80.3)	** < 0.001** ^b^
Yes	21 (1.7)	14 (19.7)	
Chronic kidney disease (%)			
No	1230 (98.3)	56 (78.9)	** < 0.001** ^b^
Yes	24 (1.7)	15 (21.1)	
Chronic liver disease (%)			
No	1236 (98.6)	61 (85.9)	** < 0.001** ^b^
Yes	18 (1.4)	10 (14.1)	
Dermatitis (%)			
No	829 (66.1)	38 (53.5)	**0.030** ^b^
Yes	425 (33.9)	33 (46.5)	
Organ transplant (%)			
No	1249 (99.6)	63 (88.7)	** < 0.001** ^b^
Yes	5 (0.4)	8 (11.3)	

^a^Kruskal-Wallis rank sum test. ^b^Pearson’s Chi-squared test

Among the included patients, 721 had none-to-mild periodontitis (stages 0–1), while 604 had moderate-to-severe periodontitis (stages 2–4) (Table 2). A total of 833 periodontal non-surgical and surgical procedures were done in 381 patients before COVID-19 infection, between January 1st, 2019, and December 31st, 2020 (Supp. Table 5). Only 187 of the patients that received periodontal care fit the criteria of stage 2–4 periodontitis by showing radiographic signs of moderate to severe interdental bone loss.

**Table 2:** Demographic characteristics of patients according to the presence and treatment of periodontitis

Conditions	No periodontitis (n = 721)	Non-treated periodontitis (*n* = 417)	Treated periodontitis (*n* = 187)	*p*
Age (median [IQR])	33.00 [25.00, 41.00]	49.00 [39.00, 59.00]	51.00 [42.50, 58.00]	** < 0.001** ^a^
BMI (median [IQR])	28.00 [24.21, 32.05]	30.01 [26.43, 33.78]	29.76 [26.86, 33.75]	** < 0.001**^a^
Sex (%)				
Female	464 (64.4)	191 (45.8)	93 (49.7)	** < 0.001**^b^
Male	257 (35.6)	226 (54.2)	94 (50.3)	
Citizenship status (%)				
Non-Qatari citizen	290 (40.2)	243 (58.3)	80 (42.8)	** < 0.001** ^b^
Qatari Citizen	431 (59.8)	174 (41.7)	107 (57.2)	
Smoking habits (%)				
Never smoker	653 (87.4)	339 (81.3)	157 (84.0)	** < 0.001** ^b^
Past/present smoker	68 (12.6)	78 (18.7)	30(16.0)	
Hypertension (%)				
No	625 (86.7)	246 (59.0)	132 (70.6)	** < 0.001** ^b^
Yes	96 (13.3)	171 (41.0)	55 (29.4)	
Diabetes (%)				
No	579 (80.3)	227 (54.4)	117 (62.6)	** < 0.001** ^b^
Yes	142 (19.7)	190 (45.6)	70 (37.4)	
Asthma (%)				
No	628 (87.1)	354 (84.9)	160 (85.6)	0.561 ^b^
Yes	93 (12.9)	63 (15.1)	27 (14.4)	
Autoimmune (%)				
No	712 (98.8)	413 (99.0)	183 (97.9)	0.488 ^b^
Yes	9 (1.2)	4 (1.0)	4 (2.1)	
Cardiovascular (%)				
No	636 (88.2)	267 (64.0)	142 (75.9)	** < 0.001** ^b^
Yes	85 (11.8)	150 (36.0)	45 (24.1)	
COPD (%)				
No	719 (99.7)	410 (98.3)	186 (99.5)	**0.029** ^b^
Yes	2 (0.3)	7 (1.7)	1 (0.5)	
Peptic ulcer (%)				
No	713 (98.9)	404 (96.9)	180 (96.3)	**0.019** ^b^
Yes	8 (1.1)	13 (3.1)	7 (3.7)	
Cancer (%)				
No	710 (98.5)	393 (94.2)	187 (100.0)	** < 0.001** ^b^
Yes	11 (1.5)	24 (5.8)	0 (0.0)	
Chronic kidney disease (%)				
No	712 (98.8)	390 (93.5)	184 (98.4)	** < 0.001** ^b^
Yes	9 (1.2)	27 (6.5)	3 (1.6)	
Chronic liver disease (%)				
No	710 (98.5)	404 (96.9)	183 (97.9)	0.198 ^b^
Yes	11 (1.5)	13 (3.1)	4 (2.1)	
Dermatitis (%)				
No	491 (68.1)	260 (62.4)	116 (62.0)	0.083 ^b^
Yes	230 (31.9)	157 (37.6)	71 (38.0)	
Organ transplant (%)				
No	718 (99.6)	407 (97.6)	187 (100.0)	**0.002** ^b^
Yes	3 (0.4)	10 (2.4)	0 (0.0)	

^a^Kruskal-Wallis rank sum test. ^b^Pearson’s Chi-squared test. The statistical analysis was testing for differences between the three groups

On average, the percentage of periodontal bone loss was 11.0 (SD 4.5) % among healthy to mild periodontitis patients (stages 0–1), 25.3 (SD 12.1) % for non-treated periodontitis (stages 2–4) and 27.3 (SD 11.6) % for treated stage 2–4 periodontitis subjects, respectively (Supp. Tables 3 & 4). Subjects with treated stage 2–4 periodontitis presented similar confounders to those with non-treated periodontitis (stages 2–4), except for cardiovascular diseases, hypertension and diabetes that were significantly more common in non-treated periodontitis (*p* < 0.05) (Table 2).

The presence of periodontitis (stages 2–4) (regardless of treatment) was associated with age, BMI, sex, citizenship status, smoking habits, hypertension, diabetes, cardiovascular diseases, COPD, peptic ulcer, cancer, chronic kidney disease and organ transplantation (Table 2). The following comorbidities were significantly more common in non-treated periodontitis group than in treated periodontitis: cancer, kidney disease, organ transplant, cardiovascular disease, diabetes, hypertension and non-citizens (*p* < 0.05; Fisher’s exact test).

Risk analysis of COVID-19 complications revealed that while periodontitis stage 2–4 (regardless of treatment) was associated with higher risk of complications (i.e. need for mechanical ventilation [AOR = 3.32, 95% CI 1.10–10.08, *p* = 0.034]) (Supp. Table 1), subjects with treated periodontitis had a lower risk than the non-treated ones. Adjusted OR analysis comparing treated and non-treated periodontitis (stages 2–4) revealed that treated patients were at lower risk of complications; however, this was not statistically significant (Supp. Table 2). On the other hand, adjusted OR analysis comparing periodontitis patients to healthy ones showed that non-treated periodontitis was associated with a significant risk of need for mechanical ventilation (AOR = 3.91; 95% CI 1.21–12.57, *p* = 0.034, while for treated periodontitis, the risk was not significant (AOR = 1.28; 95% CI 0.25–6.58, *p* = 0.768) (Table 3).

**Table 3:** Risk analysis of COVID-19 complications (any complication, ICU admission, ventilation and death) associated with treated periodontitis and untreated periodontitis

Periodontal condition	COVID-19 complications		OR (95% CI)	AOR (95% CI)*	Adjusted p
	None	Any			
No periodontitis	698	23	1	1	
Non-treated periodontitis	375	42	3.40 (2.01–5.74)	1.92 (0.96–3.83)	0.065
Treated periodontitis	181	6	1.01 (0.40–2.51)	1.39 (0.48–4.04)	0.543
	None	Ventilation			
No periodontitis	698	6	1	1	
Non-treated periodontitis	375	23	7.14 (2.88–17.68)	**3.91 (1.21–12.57)**	**0.022**
Treated periodontitis	181	3	1.93 (0.48–7.78)	1.28 (0.25–6.58)	0.768
	None	Deceased			
No periodontitis	698	5	1	1	
Non-treated periodontitis	375	13	4.84 (1.71–13.68)	3.56 (0.55–22.73)	0.181
Treated periodontitis	181	1	0.77 (0.09–6.64)	1.13 (0.07–18.43)	0.993
	None	ICU			
No periodontitis	698	21	1	1	
Non-treated periodontitis	375	35	3.10 (1.78–5.41)	1.81 (0.88–3.69)	0.104
Treated periodontitis	181	6	1.10 (0.44–2.77)	1.31 (0.45–3.81)	0.618

^*^Adjusted to sex, age, diabetes, smoking habits, BMI and co-morbidities

**Fig. 1:**
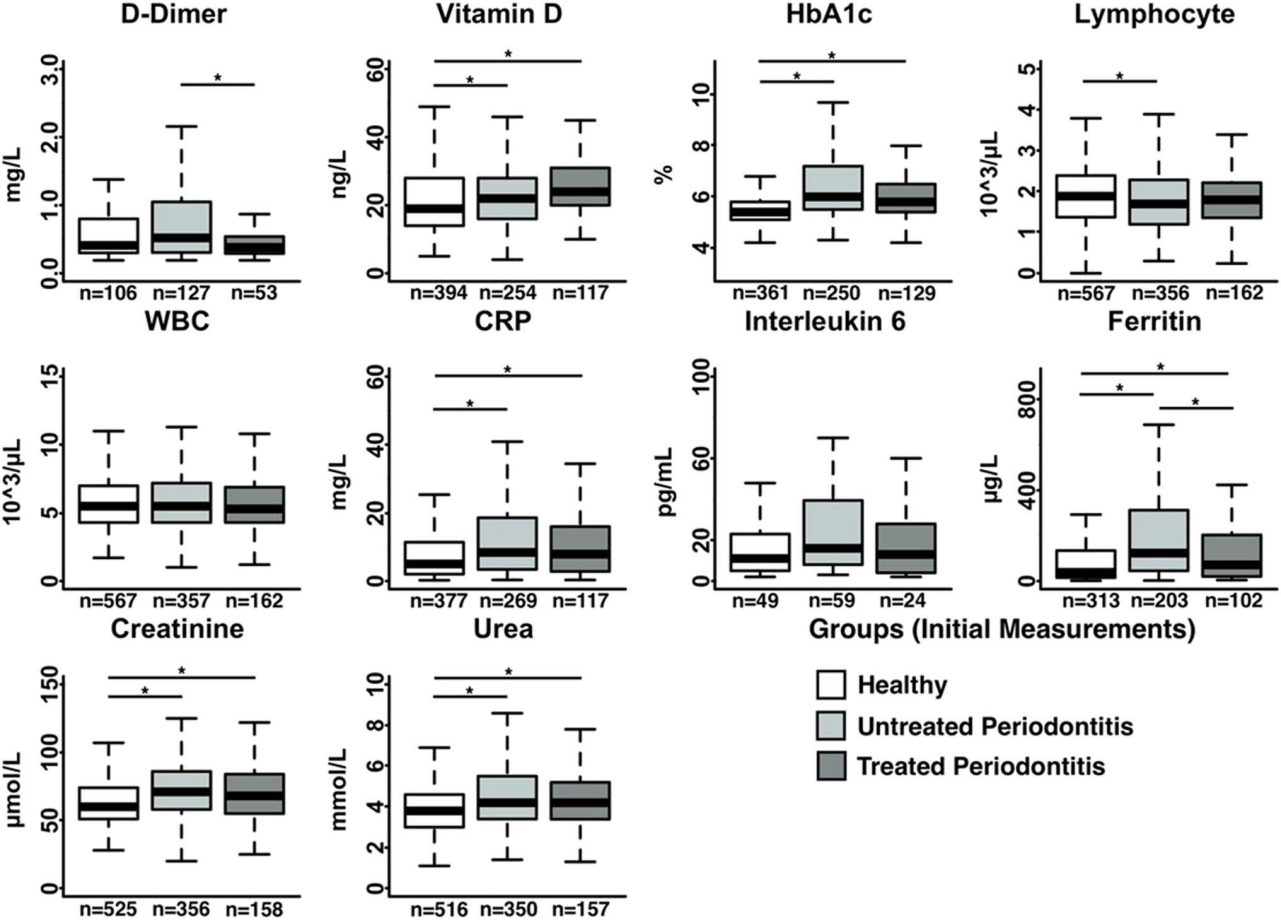
Box plots of the blood work of COVID-19 patients at admission (initial) comparing patients without periodontitis with those with periodontitis that have received treatment for periodontitis and those that did not receive periodontal treatment. (*) indicates significant differences between groups (*p* < 0.05). The letter n indicates the number of patients analysed for each parameter and condition

**Fig. 2:**
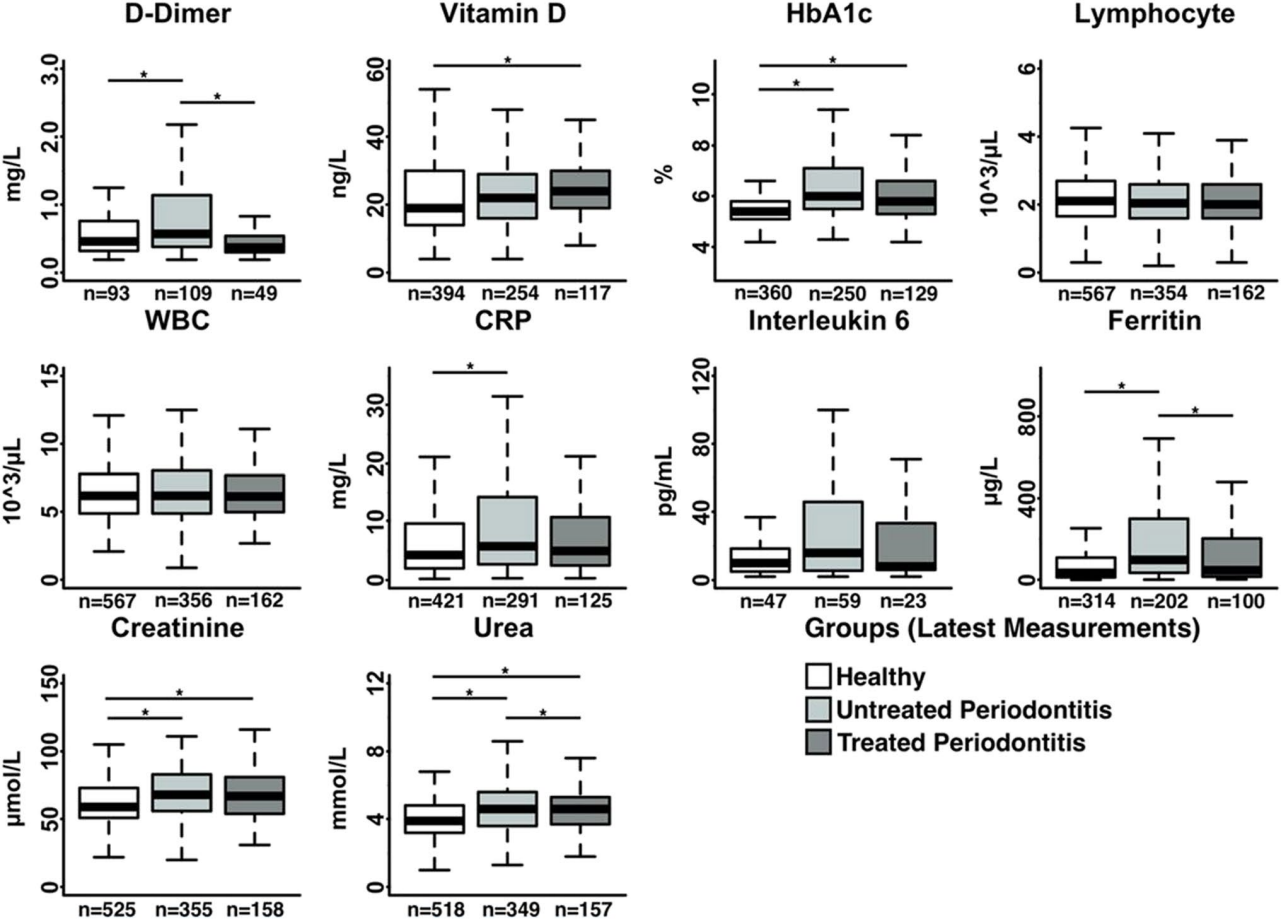
Box plots of the blood work of COVID-19 patients before being discharged, comparing patients without periodontitis with those with periodontitis that have received treatment for periodontitis and those that did not receive periodontal treatment. (*) indicates significant differences between groups (*p* < 0.05). The letter n indicates the number of patients analysed for each parameter and condition

Figures 1 and 2 depict the blood biomarkers analyses comparing periodontally healthy (stage 0–1 periodontitis), non-treated periodontitis (stages 2–4), and treated periodontitis stages 2–4 subjects at admission and discharge, respectively. Compared to periodontally healthy patients, periodontitis stage 2–4 patients (both treated and non-treated) had significantly higher levels of creatine, urea, and HbA1c, throughout the course of their COVID-19 disease as well as higher levels of ferritin, vitamin D, and CRP at the initial stage of COVID-19. Compared to periodontally healthy patients, non-treated periodontitis (stage 2–4) subjects showed significantly lower levels of lymphocytes upon admission and higher levels of D-dimer, CRP and ferritin before discharge.

Furthermore, subjects with treated periodontitis (stages 2–4) demonstrated significantly lower levels of D-dimer and ferritin compared to non-treated periodontitis patients throughout the course of their COVID-19 disease, and lower levels of urea at the later stage of the disease. Other biomarkers such as HbA1c, and IL-6 showed higher levels in non-treated periodontitis when compared to treated subjects, but these differences were not significant.

Among the 110 patients that underwent bacterial culture from respiratory sites, only 9 had positive cultures, 5 of whom were also periodontitis subjects. However, there was no significant association between the periodontitis and positive bacterial cultures (OR = 0.79 95% CI 0.20–3.11; *p* = 0.731). The bacterial species identified were *Stenotrophomonas maltophilia, Staphylococcus hominis, Staphylococcus epidermidis, Pseudomonas putida* and *Klebsiella pneumoniae*. Cases with positive cultures for *Stenotrophomonas maltophilia* (*n* = 2) and *Staphylococcus hominis* (*n* = 1) corresponded to patients without periodontitis; *Staphylococcus epidermidis* (*n* = 3) and *Klebsiella pneumoniae* (*n* = 1) were only found in patients with non-treated periodontitis and *Pseudomonas putida* (*n* = 1) in a patient with treated periodontitis. Among these 9 patients with bacterial pulmonary infections, 7 were admitted to the ICU, 6 needed mechanical ventilation, and 4 died.

## Discussion

This study revealed that, compared to periodontally healthy patients, patients with moderate to advanced periodontitis (stages 2–4) who did not receive periodontal therapy could be at higher risk of needing mechanical ventilation. On the other hand, periodontitis patients that received periodontal care were not associated with increased risk of COVID-19 complications. Diagnosis of periodontitis (stages 2–4) was associated with increased levels of serum biomarkers of inflammation, coagulation, diabetes and kidney dysfunction even among treated patients. However, compared to non-treated periodontitis subjects, treated periodontitis patients had significantly lower levels of D-dimer and ferritin throughout the course of the disease and lower levels of urea prior to discharge.

The significant association between non-treated periodontitis and mechanical ventilation corroborates previous reports linking periodontitis to COVID-19 complications. A previous study demonstrated a significant association between periodontitis and the need for mechanical ventilation (AOR = 4.57, 95% CI 1.19–17.4) [11], and other studies based on phone surveys found an association between periodontitis and COVID-19-related hospital admissions and mortality [22]. However, this study presents evidence on the possible impact of periodontal therapy on COVID-19 complications, adding more evidence to the already known effect of periodontal therapy on systemic conditions such as diabetes and cardiovascular diseases [23].

Periodontitis and severe COVID-19 share several common risk factors such as age, obesity, diabetes and hypertension, among others [23–25]. Therefore, it may be argued that these predisposing conditions could be behind the association between periodontitis and unfavourable COVID-19 outcomes. However, the findings of this study strengthen the hypothesis linking COVID-19 complications directly to periodontitis.

In this study, patients with non-treated moderate-to-advanced periodontitis (stages 2–4) presented higher risk of COVID-19-related ICU admissions and death; however, these associations were not significant. This contradicts previous findings demonstrating a significant association of periodontitis with COVID-19-related deaths (AOR = 8.81, 95% CI 1.00–77.7) and ICU admissions (AOR = 3.54, 95% CI 1.39–9.05)[11]. This could be explained by the changes in the lethality of virus variants across the course of the pandemic. While our first study was limited to the first and deadliest COVID-19 wave in Qatar, the present study covers an extended observational period of the pandemic that includes a second phase of the pandemic that was characterized by lower lethality [26]. Accordingly, the proportionally smaller number of deaths in this second study limited our ability to analyse the risk of death associated with periodontitis. This observation also implies that the nature of the SARS-CoV2 variants and COVID-19 waves could indeed play a role in the association between periodontitis and COVID-19 severity.

Sub-analysis of COVID-19 complications in our study was necessary because COVID-19 deaths are uncommon and ICU admission may depend on physicians’ subjective evaluations [27, 28]. This is particularly relevant in the context of Qatar because due to its young population and well-functioning healthcare system it had one of the lowest rates of COVID-19 complication in the world [28]. This implies that in other populations with higher rates of COVID-19 complications, periodontal health might have a different impact.

In this study, regardless of periodontal therapy, patients with periodontitis presented higher blood levels of inflammatory biomarkers than those without periodontitis. This corroborates different investigations showing that periodontitis is associated with systemic inflammation biomarkers such as CRP, IL-1 and IL-6 [23, 29, 30]. Since COVID-19 complications are associated with the same biomarkers [31], it has been hypothesized that periodontitis could influence COVID-19 outcomes through its impact on systemic inflammation by priming the immune system to an exacerbated reaction to COVID-19 [32]. It has also been proposed that viral infections could play a role in the association between periodontitis and systemic conditions through their ability to exacerbate inflammation, compromise the immune responses and synergize with bacteria [33]. For instance, viruses from the herpes and human papillomavirus families cause primary viral infections in the oral cavity, and two herpesviruses, Epstein‐Barr virus and cytomegalovirus, have been associated with marginal periodontitis and the inflammatory process of periapical bone destruction [34, 35].

Interestingly, in the current study, ferritin, an indicator of systemic inflammation, presented lower serum levels in patients with treated periodontitis compared to non-treated periodontitis. A similar observation was made on the levels of CRP before patient discharge. This could confirm that the impact of periodontitis and periodontal care on COVID-19 complications could be mediated by inflammatory pathways.

D-dimer, a coagulation biomarker indicative of thrombosis, presented significantly lower levels in treated than in non-treated periodontitis patients. This seems to suggest that coagulopathy may also be linking COVID-19 complications with periodontitis and that periodontitis could be exacerbating COVID-19 coagulopathies as hypothesized by Lloyd-Jones et al. [36], although further research would be needed to confirm this.

Periodontitis has a well-established association with coagulopathies. Periodontitis has been associated with platelet activation [37], and periodontal care has been shown to improve endothelial function and coagulation disorders [38] and reduce the levels of D-dimer [39]. On the other hand, D-dimer levels are known to increase in COVID-19 patients, as a reflection of coagulation activation caused by viremia, inflammation, superinfection and/or organ dysfunction, and they are directly correlated to COVID-19 complications and mortality [40].

Another possible mechanism linking periodontitis with COVID-19 severity may be the well-established association between oral health and respiratory diseases [41–43]. The oral cavity is a reservoir for respiratory pathogens [43, 44], and periodontitis and poor oral hygiene have been associated with increased risk of pneumonia [45], while periodontal care has been shown to prevent deaths from pneumonia in high-risk patients [46]. Thus, it has been hypothesized that periodontitis could aggravate COVID-19 by increasing the risk of direct oral bacteria aspiration or inoculation into the lower respiratory tract [41, 47]. In this study, out of the 110 patients that underwent lung swaps, only nine presented positive bacterial cultures, and among them, only five had signs of periodontitis, indicating no significant association between periodontitis and the risk of pulmonary bacterial infections among this group of COVID-19 patients. The limited number of patients with microbial testing from respiratory sites prevented us from drawing clear conclusions on the links between periodontal microbes and secondary lung infections in COVID-19 patients. Nevertheless, the bacteria species identified (*Stenotrophomonas maltophilia*, and *Staphylococcus hominis*, *Staphylococcus epidermidis, Pseudomonas putida a*nd *Klebsiella pneumoniae*) are all known to be present in the oral cavity [48–54]. In this study, *Stenotrophomonas maltophilia*, an antibiotic resistant opportunistic bacteria found in the oral cavity of immunocompromised patients [53, 54], was only identified in patients without periodontitis. Similarly, *Staphylococcus hominis* was only found in patients without periodontitis, although this bacterium could be found in periodontitis and periapical lesions [51, 52]. In contrast, *Staphylococcus epidermidis* (*n* = 3), *Pseudomonas putida* (*n* = 1) and *Klebsiella pneumoniae* (*n* = 1) were only found in patients with periodontitis. *Staphylococcus epidermidis* are found in the oral cavity, especially in patients with periodontitis [51], and *Klebsiella pneumoniae* are found in periodontal pockets [50]. The small incidence of bacterial infections reported in this study seems to indicate that aspiration of periodontal pathogens to the lung probably plays a minor role in the association between periodontitis and COVID-19 complications. However, even though these numbers were small, the mortality associated with these cases was extremely high (4 out of the 9 patients with positive cultures died), amounting to 21% of all registered fatalities.

The national COVID-19 protocols implemented during the study period included the prophylactic administration of azithromycin for hospitalized COVID-19 patients. As a result of this policy, almost 80% of the patients hospitalized in our study received azithromycin. This antibiotic is known to have beneficial effects against periodontitis [55], and thus it could be hypothesized that periodontitis patients would have benefited from it. However, future studies would be needed to investigate this possibility.

One of the limitations of this observational study is that the periodontal clinical examination of COVID-19 patients was not feasible, and the use of radiographs and dental records was the best available option for assessing periodontal status and effectiveness of periodontal treatments prior to COVID-19 infection. Nonetheless, previous studies have shown that radiological bone loss is a good indicator for periodontitis [56], and we were able to validate our assessments using the subset of patients registered at the Division of Periodontics at HMC.

Various socioeconomic factors such as education, income and willingness to contact medical and dental providers could also be confounders that might have an impact on oral diseases and COVID-19 severity. In our study, we were not able to obtain direct information on these parameters, and this is a limitation. However, we were able to analyse and adjust for an indirect socioeconomic indicator, which is the citizenship status, a strong indicator of household income in Qatar. A similar approach has been used in previous COVID-19 studies in Qatar [57]. Our analyses showed that Qatari citizens had lower incidence of periodontitis and higher rates of periodontal treatment, probably due to their access to preventive dental care programs. However, citizenship was not independently associated with higher risk of COVID-19 complications, probably because the non-citizenship segment of the population is relatively young in age, and all COVID-19 patients received free access to medical care.

Another limitation was that periodontal therapy was not randomized; hence, differences between treated and non-treated groups might not be solely attributed to the interventions but could also result from other unaccounted factors. Indeed, periodontal therapy was significantly associated with some confounders (Table 2), although our statistical analysis adjusted for this.

This study was conducted on non-vaccinated subjects and the data was collected before COVID-19 vaccines became available to the subjects; thus, the associations observed might be different in vaccinated patients.

Despite these limitations, the reported findings have important clinical implications. The fact that patients with treated periodontitis were less likely to suffer COVID-19 complications than non-treated ones further strengthens the hypothesis linking periodontitis to COVID-19 complications and suggests that managing periodontitis could help reduce the risk for COVID-19 complications, although future research is needed to verify this.

In conclusion, this observational study showed that COVID-19 patients with non-treated periodontitis (stages 2–4) were significantly more likely to need mechanical ventilation; however, this risk was not significant for patients with treated periodontitis. Increased blood levels of D-dimer and ferritin in patients with non-treated periodontitis compared to periodontally healthy and treated periodontitis patients could imply that periodontitis increases the risk of COVID-19 complications through its detrimental effects on inflammation and coagulation.

## Supplementary information

Below is the link to the electronic supplementary material.

Supplementary file1 (DOCX 20 KB)
