# Supplementary material for: Association of periodontal therapy, with inflammatory biomarkers and complications in COVID-19 patients: a case control study
Source: Clin Oral Investig. 2022 Jul 29;26(11):6721–32. doi: 10.1007/s00784-022-04631-6 (PMC9643194; doi:10.1007/s00784-022-04631-6)
Supplement: Supplementary file 1 — Supplementary file1 (DOCX 20 KB) [file 784_2022_4631_MOESM1_ESM.docx]

**Association of periodontal therapy, with inflammatory biomarkers and complications in COVID-19 patients: A case control study**

**Journal of Clinical Oral Investigation**

Khalid N. Said^1^, Ahmed M. Al-Momani^1^, Jassim A. Almaseeh^1^, Nadya Marouf^1^, Amer Shatta^1^, Jassim Al-Abdulla^1^, Sahar Alaji^1^, Hanin Daas^2^, Shailaja S. Tharupeedikayil^1^, Venkateswara Rao Chinta^3^, Ali Ait Hssain^4^, Mohammad Abusamak^5^, Shiraz Salih^2^, Noha Barhom^2^, Wenji Cai^5^, Mariano Sanz^6^, Faleh Tamimi ^2*^.

^1^ Department of Dentistry and Oral Health Institute, Hamad Medical Corporation, Doha, Qatar.

^2^ College of Dental Medicine, QU Health, Qatar University, Doha, Qatar

^3^ Department of Business Intelligence, Hamad Medical Corporation, Doha, Qatar

^4^ Medical Intensive Care Unit, Hamad Medical Corporation, Doha, Qatar

^5^ Faculty of Dentistry, McGill University, Montreal, Canada

^6^ Faculty of Odontology, ETEP Research Group, Universidad Complutense de Madrid, Madrid, Spain

**Correspondence:** Dr. Faleh Tamimi

College of Dental Medicine, QU Health, Qatar University, Doha, Qatar

Email: [fmarino@qu.edu.qa](mailto:fmarino@qu.edu.qa)

Supplementary Table 1: Comparison of complications between patients with no periodontitis and patients with periodontitis (regardless of history of treatment)

| Complications | | Periodontitis | | OR(95% CI) | P | AOR(95% CI) | P |
| --- | --- | --- | --- | --- | --- | --- | --- |
|  |  | no | yes |  |  |  |  |
| Deceased | no | 698 | 556 | 1 |  | 1 |  |
|  | yes | 5 | 14 | 3.51(1.26-9.82) | 0.018 | 3.56(0.60-21.28) | 0.163 |
| ICU admission | no | 698 | 556 | 1 |  |  |  |
|  | yes | 21 | 41 | 2.45(1.43-4.19) | <0.001 | 1.76(0.89-3.46) | 0.101 |
| Ventilation | no | 698 | 556 |  |  | 1 |  |
|  | yes | 6 | 26 | 5.44(2.22-13.30) | <0.001 | **3.32(1.10-10.08)** | **0.034** |
| Any complications | no | 698 | 556 | 1 |  | 1 |  |
|  | yes | 23 | 48 | 2.62(1.57-4.36) | <0.001 | 1.81(0.94-3.48) | 0.078 |

*Adjusted to sex, age, diabetes, smoking habits, BMI and other co-morbidities

Supplemental Table 2: Direct comparison between treated and untreated periodontitis: complications

| Complications | | Periodontal Therapy | | OR (95%CI) | P fishers’ exact test | AOR(95%CI) | p |
| --- | --- | --- | --- | --- | --- | --- | --- |
|  |  | no | yes |  |  |  |  |
| Deceased | no | 375 | 181 |  |  |  |  |
|  | yes | 13 | 1 | 0.16(0.02-1.23) | **0.045** | 0.41(0.04-3.74) | 0.425 |
| Ventilation | no | 375 | 181 |  |  |  |  |
|  | yes | 23 | 3 | 0.27(.08-.91) | **0.029** | 0.41(0.11-1.47) | 0.170 |
| ICU | no | 375 | 181 |  |  |  |  |
|  | yes | 35 | 6 | 0.36(0.15-0.86) | **0.015** | 0.52(0.21-1.32) | 0.170 |
| Any complication | no | 375 | 181 |  |  |  |  |
|  | yes | 42 | 6 | 0.30(0.12-0.71) | **0.003** | 0.47(0.189-1.19) | 0.101 |

*Adjusted to sex, age, diabetes, smoking habits, BMI and co-morbidities

Supplementary Table 3. Severity of periodontitis according to the diagnosis and treatment of periodontitis

| Periodontal status | Percentage of bone loss in worst site (%) |
| --- | --- |
| Periodontally healthy (stage 0-1) | 11.0 SD 4.5 ^c, b^ |
| Untreated periodontitis (stage 2-4) | 25.3 SD 12.1 ^a, c^ |
| Treated periodontitis (stage 2-4) | 27.3 SD 11.6 ^a, b^ |

^a^ Significantly different from periodontally healthy (p<0.05), ^b^ significant different from Untreated periodontitis (p<0.05), ^c^ significantly different from treated periodontitis

Supplementary Table 4. Severity of periodontitis according to COVID-19 complications

| COVID-19 complication | Percentage of root exposure in worst site |
| --- | --- |
| No complication | 17.4 SD11.0 |
| Death | 29.4 SD23.8 ^a^ |
| ICU | 21.9 SD 11.3 ^a^ |
| Ventilation | 24.1 SD 9.5 ^a^ |
| Any complication | 23.8 SD 16.2 ^a^ |

^a^ Significantly different from patients with no complications (p<0.05) (one way ANOVA with Post hoc Bonferroni test)

Supplementary Table 5. Periodontal treatments provided before COVID-infection between January 1^st^ 2019 and December 31^st^ 2020

| Periodontal procedure | number |
| --- | --- |
| Scaling, root planing and polishing | 779 |
| Regenerative- Bone graft surgery | 39 |
| Gingivectomy surgery | 6 |
| Periodontal flap procedure | 9 |
| Total | 833 |

Supplementary Table 6: Validation of comorbidity index

|  | | COVID-19 complications | | OR(95%CI) | p |
| --- | --- | --- | --- | --- | --- |
|  |  | no | yes |  |  |
| Number of comorbidities | 0 | 603 | 6 | 1 |  |
|  | 1 | 310 | 16 | 5.19(2.01-13.39) | <0.001 |
|  | 2 | 341 | 49 | 14.44(6.12-34.06) | <0.001 |
